# Association of increasing gross tumor volume dose with tumor volume reduction and local control in fractionated stereotactic radiosurgery for unresected brain metastases

**DOI:** 10.1186/s13014-024-02487-6

**Published:** 2024-07-27

**Authors:** Naoyuki Kanayama, Toshiki Ikawa, Koji Takano, Hideyuki Arita, Masahiro Morimoto, Takero Hirata, Kazuhiko Ogawa, Teruki Teshima, Koji Konishi

**Affiliations:** 1 Department of Radiation Oncology, Osaka International Center Institute, 3-1-69 Otemae, Chuo- ku, Osaka-shi, 541-8567 Osaka Japan; 2 Department of Neurosurgery, Osaka International Center Institute, 3-1-69 Otemae, Chuo-ku, Osaka-shi, 541-8567 Osaka Japan; 3 Department of Radiation Oncology, Osaka University Graduate School of Medicine, 2-2 Yamadaoka, Suita-shi, 565-0871 Osaka Japan; 4 Osaka Heavy Ion Therapy Center, 3-1-10 Otemae, Chuo-ku, Osaka, Japan

**Keywords:** Inhomogeneous dose distribution, Volumetric analysis, Volume reduction, Local control, Fractionated stereotactic radiosurgery, Brain metastases

## Abstract

**Background:**

Fractionated stereotactic radiosurgery (fSRS) is an important treatment strategy for unresected brain metastases. We previously reported that a good volumetric response 6 months after fSRS can be the first step for local control. Few studies have reported the association between gross tumor volume (GTV) dose, volumetric response, and local control in patients treated with the same number of fractions. Therefore, in this study, we aimed to investigate the GTV dose and volumetric response 6 months after fSRS in five daily fractions and identify the predictive GTV dose for local failure (LF) for unresected brain metastasis.

**Methods:**

This retrospective study included 115 patients with 241 unresected brain metastases treated using fSRS in five daily fractions at our hospital between January 2013 and April 2022. The median prescription dose was 35 Gy (range, 30–35 Gy) in five fractions. The median follow-up time after fSRS was 16 months (range, 7–66 months).

**Results:**

GTV D80 > 42 Gy and GTV D98 > 39 Gy were prognostic factors for over 65% volume reduction (odds ratio, 3.68, *p <* 0.01; odds ratio, 4.68, *p <* 0.01, respectively). GTV D80 > 42 Gy was also a prognostic factor for LF (hazard ratio, 0.37; *p* = 0.01).

**Conclusions:**

GTV D80 > 42 Gy in five fractions led to better volume reduction and local control. The goal of planning an inhomogeneous dose distribution for fSRS in brain metastases may be to increase the GTV D80 and GTV D98. Further studies on inhomogeneous dose distributions are required.

**Supplementary Information:**

The online version contains supplementary material available at 10.1186/s13014-024-02487-6.

## Background

Stereotactic radiosurgery (SRS) and fractionated stereotactic radiosurgery (fSRS) are essential treatment strategies for brain metastases [1, 2]. The clinical application of fSRS is increasing with advancements in linear accelerator (Linac) and radiation technologies [3]. Various radiation doses and fractions have been used in fSRS for brain metastases [1, 4, 5] and different formulas have been used to calculate the biological effective dose for fSRS [4–8]. These inconsistencies result in different doses calculated for tumor control. The same number of fractions is preferable when discussing tumor control and radiotherapy doses. However, only a few reports discuss tumor control in the same number of treatment fractions using Linac fSRS for unresected brain metastases [3, 9–14].

As per the Response Assessment in Neuro-Oncology Brain Metastases (RANO-BM) guidelines, the assessment and reporting of volumetric measurements are important, and their inclusion as a study endpoint should be encouraged [15] as volumetric measurements have lower variability than linear measurements [16]. However, few studies use volumetric measurements owing to the associated complexity [8, 15, 17, 18].

We previously reported over 65% and over 90% volume reductions 6 months after fSRS and SRS predicted local control [19]. Few studies have reported the association between gross tumor volume (GTV) dose and volumetric response, and local control for fSRS with the same number of fractions [9]. Therefore, in this study, we aimed to identify the prognostic GTV dose for volumetric response—specifically, over 65% reduction (O65R) and over 90% reduction (O90R) in volume—of brain metastases 6 months after fSRS in five fractions, and identify the prognostic GTV dose for local failure (LF).

## Methods

### Patients

Overall, 115 patients with 241 unresected brain metastases treated by fSRS in five daily fractions at our institute between January 2013 and April 2022 were included in this study. Patient data were retrospectively collected from our electronic database. The inclusion criteria were unresected brain metastases treated by five daily fractions at our institute. The exclusion criteria were the following: brain metastases < 0.3 cc at baseline, no magnetic resonance imaging (MRI) 5.0–8.5 months after fSRS, whole-brain radiotherapy before MRI evaluation, and brain metastases with local tumor progression before MRI evaluation, performed approximately 6 months (median, 6.2 months; range, 5.0–8.3 months) after fSRS.

The patient characteristics and details of the brain metastases and fSRS are presented in Table 1. A total of 179 brain metastases treated with systemic therapy concurrently (within 1 month before or after fSRS); 33 treated with immunotherapy, including immune checkpoint inhibitors; and 88 treated with target therapy, including angiogenesis inhibitors, anti-human epidermal growth factor receptor type 2 antibodies, anti-epidermal growth factor receptor antibodies, tyrosine kinase inhibitors, serine/threonine kinase inhibitors, and cyclin-dependent kinase inhibitors.

**Table 1:** Patient, brain metastases, and fSRS characteristics

		*n*	(%)
Patient characteristics			
Total		115	
Age (years)	Median (range)	66 (22–85)	
	22–65	56	(48.7)
	> 65	59	(51.3)
Sex	Male	55	(47.8)
	Female	60	(52.2)
PS	0	70	(60.9)
	1	34	(29.6)
	2	10	(8.7)
	3	1	(0.9)
Primary cancer	Lung	71	(61.7)
	Breast	14	(12.2)
	GI	6	(5.2)
	Kidney	3	(2.6)
	Melanoma	5	(4.3)
	Others	16	(13.9)
Brain metastases and fSRS characteristics			
Total		241	
Prescription dose	30 Gy/5 fr	28	(11.6)
	35 Gy/5 fr	213	(88.4)
Each GTV (cc)	Median (range)	1.1 (0.3–33.1)	
	0.3–1	116	(48.1)
	> 1–4	70	(29.0)
	> 4	55	(22.8)

*Abbreviations* fSRS = fractionated stereotactic radiosurgery; PS = performance status; GI = gastrointestinal; PTV = planning target volume; GTV = gross tumor volume; fr = fraction

### Treatments

The fSRS treatment has been described previously [19–21]. All patients were immobilized using a thermoplastic mask, and planning computed tomography was performed using an iodine-based contrast agent. The GTV was delineated using T1-weighted gadolinium-enhanced MRI. An isotropic margin of 1 mm (range, 1–3 mm) was applied to the GTV to obtain the planning target volume (PTV).

The median prescription dose was 35 Gy (range, 30–35 Gy) in five daily fractions on weekdays. The dose was prescribed to cover 95% or 99% of the combined PTVs. The median isodose line (prescription dose/maximum dose for GTV) was 52% (range, 40–95%). An inhomogeneous dose distribution was allowed. The doses to the brain tissue were reduced to a minimum during the optimization process. Automated non-coplanar volumetric-modulated arc therapy (VMAT) (HyperArc; Varian Medical Systems, Palo Alto, CA), coplanar VMAT, or dynamic conformal arc therapy with C-arm Linac (Clinac 23Ex, Ture Beam STX, or Edge; Varian Medical Systems, Palo Alto, CA, USA) was used for fSRS.

Follow-ups included clinical examination and MRI. Brain MRI every 3 months was recommended. The interval was shortened when the tumor volume was increased or new symptoms developed. The median follow-up time after fSRS was 16 months (range, 7–66 months).

The evaluated MRIs were imported into the radiotherapy planning system. The tumor volume was delineated by a radiation oncologist, including the tumor and the fSRS effects. The tumor volume reduction rate from GTV was evaluated.

### Definitions

LF was calculated from the time of MRI evaluation to the radiological observation of tumor progression in a treated lesion. Tumor progression was defined according to the RANO-BM guidelines [15]. The definition of tumor progression was applied to each brain metastasis. The differential diagnoses of tumor progression and brain necrosis have been described previously [19]. We used adverse radiation effect (ARE) to capture all cases of radiation necrosis. The definition of ARE is consistent with the prior report by Sneed et al. [22]. ARE was calculated from the first day of fSRS.

### Statistical analyses

LF and ARE were estimated using the cumulative incidence function with death as a competing risk. Univariate and multivariate analyses of factors associated with LF and ARE were performed using the Fine–Gray model to determine hazard ratios (HRs). Univariate and multivariate analyses of the factors associated with O65R and O90R were performed using a logistic regression model to determine the odds ratios (ORs). Furthermore, GTV D98, D80, D60, D40, D20, and D2 were analyzed. Pearson’s correlation coefficient (*r*) was used to evaluate the correlation between the GTV parameters. Moreover, Spearman’s correlation coefficient (*ρ*) was used to evaluate the correlation between the clinical and GTV parameters. The Kendall rank correlation coefficient (*τ*) was used to evaluate the correlation between binary variables. When (*r*), (*ρ*), and (*τ*) were > 0.60, only one variable was used for the multivariate analysis. The Youden index was used to identify the optimal threshold value for volume reduction. The lowest Akaike information criterion (AICc) value was considered the most predictive. Evidence ratios (EVRs) were calculated, and models with an EVR < 2.7 were considered to have substantial support [23]. We assessed the variance inflation factor (VIF) testing for multicollinearity in the multivariate analysis of O65R and O90R. Statistical significance was set at a *p*-value of < 0.05. The statistical analyses were performed using SPSS version 25 (SPSS Inc., Chicago, IL, USA) and EZR (Saitama Medical Center, Jichi Medical University, Saitama, Japan).

## Results

### Volume reduction and LF

**Fig. 1:**
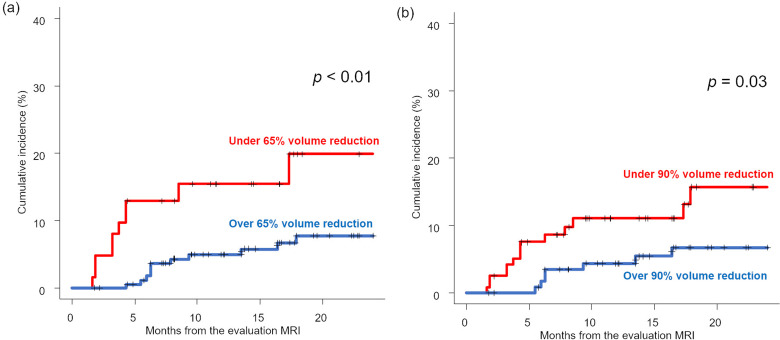
Local failure rate comparisons based on MRI findings. (**a**) Under vs. over 65% volume reduction; (**b**) under vs. over 90% volume reduction. MRI = magnetic resonance imaging

The LF at 0.5 and 1.5 years from the MRI evaluation (approximately 1 and 2 years from fSRS) for O65R was 1.80 and 7.80%, respectively (Fig. 1). The results of univariate and multivariate analyses of LF are presented in Table 2. The multivariate analysis revealed that O65R and O90R were prognostic factors for LF (HR, 0.35, *p* < 0.01 and HR, 0.41, *p* = 0.04, respectively) (Table 2).

**Table 2:** Univariate and multivariate analyses results of local failure from MRI evaluation

		Univariate			Multivariate			Multivariate			Multivariate			Multivariate		
		HR	(95% CI)	*p*-value	HR	(95% CI)	*p*-value	HR	(95% CI)	*p*-value	HR	(95% CI)	*p*-value	HR	(95% CI)	*p*-value
Over 65% volume reduction at MRI evaluation	No	1		< 0.01	1		< 0.01									
	Yes	0.33	(0.15–0.74)		0.35	(0.16–0.76)										
Over 90% volume reduction at MRI evaluation	No	1		0.03				1		0.04						
	Yes	0.38	(0.16–0.91)					0.41	(0.17–0.97)							
each GTV (cc)	0.3–1	1		< 0.01	1		< 0.01	1		< 0.01	1		0.02	1		0.01
	> 1	4.62	(1.56–13.68)		4.48	(1.50–13.37)		4.37	(1.48–12.88)		3.72	(1.28–10.83)		4.12	(1.39–12.25)	
Age (years)	22–65	1		0.70												
	> 65	0.84	(0.35–2.00)													
PS	0–1	1		0.17												
	2–3	1.80	(0.78–4.17)													
Primary cancer	Lung and breast	1		0.66												
	Others	1.23	(0.49–3.09)													
GTV dose	D80 < 42 Gy	1		< 0.01							1		0.01			
	D80 > 42 Gy	0.27	(0.12–0.61)								0.37	(0.17–0.81)				
GTV dose	D98 < 39 Gy	1		0.04										1		0.22
	D98 > 39 Gy	0.40	(0.17–0.96)											0.58	(0.25–1.38)	

*Abbreviations* MRI = magnetic resonance imaging; HR = hazard ratio; CI = confidence interval; GTV = gross tumor volume; PS = performance status

### Analysis of volume reduction

All correlations between the GTV parameters had (*r*) > 0.60. All correlations between the clinical and GTV parameters had (*ρ*) < 0.60 and (*τ*) < 0.60. Univariate analyses for O65R and O90R are presented in Supplementary Table 1.

Models that included GTV D80, age, and time for the 6-month MRI had the lowest AICc values for O65R (Supplementary Table 2). Overall, six models had EVR < 2.7 for O65R (Supplementary Table 2). Of the six models with EVR < 2.7 for O65R, five included GTV D80 and one included GTV D98 (Supplementary Table 2). Details of the multivariate analysis, including age, time for MRI evaluation, and GTV D80 or GTV D98, are presented in Supplementary Table 3; no collinearity was observed in each model. Both GTV D80 and GTV D98 were significant predictive factors for O65R (Supplementary Table 3).

Models including GTV D80, age, primary cancer, and time for MRI evaluation had the lowest AICc values for O90R (Supplementary Table 2). In addition to the lowest AICc model, the only model that included GTV D98 had EVR < 2.7 for O90R (Supplementary Table 2). Details of the multivariate analyses, including age, primary cancer, time for MRI evaluation, and GTV D80 or GTV D98, are presented in Supplementary Table 3; no collinearity was observed in any of the models (Supplementary Table 3). Both GTV D80 and GTV D98 were significant predictive factors for O90R (Supplementary Table 3).

We defined 42 Gy for GTV D80 and 39 Gy for GTV D98 as the thresholds for O65R and O90R. A strong correlation was observed between GTV D80 > 42 Gy and D98 > 39 Gy (*τ* = 0.88). The multivariate analysis revealed GTV D80 > 42 Gy and D98 > 39 Gy as predictive factors for O65R (OR, 3.68, *p* < 0.01 and OR, 4.68, *p* < 0.01, respectively; Table 3). Furthermore, GTV D80 > 42 Gy and D98 > 39 Gy were predictive factors for O90R (OR, 4.70, *p* < 0.01 and OR, 6.41, *p* < 0.01, respectively; Table 3).

**Table 3:** Multivariate analysis results for over 65% and 90% volume reduction at MRI evaluation

		Over 65% volume reduction				Over 65% volume reduction				Over 90% volume reduction				Over 90% volume reduction			
		OR	(95% CI)	*p*-value	VIF	OR	(95% CI)	*p*-value	VIF	OR	(95% CI)	*p*-value	VIF	OR	(95% CI)	*p*-value	VIF
GTV dose	D80 < 42 Gy	1		< 0.01	1.03					1		< 0.01	1.05				
	D80 > 42 Gy	3.68	(1.85–7.34)							4.70	(2.31–9.56)						
GTV dose	D98 < 39 Gy					1		< 0.01	1.03					1		< 0.01	1.06
	D98 > 39 Gy					4.68	(2.29–9.56)							6.41	(2.91–14.15)		
Age (years)	22–65	1		0.01	1.04	1		0.01	1.03	1		0.06	1.05	1		0.05	1.05
	> 65	0.45	(0.24–0.85)			0.43	(0.23–0.83)			0.58	(0.33–1.02)			0.57	(0.32–1.00)		
Primary cancer	Lung and Breast									1		0.02	1.04	1		0.01	1.05
	Others									0.46	(0.24–0.88)			0.42	(0.22–0.81)		
Time for MRI evaluation	5–6.5 months	1		0.08	1.01	1		0.14	1.01	1		< 0.01	1.02	1		0.02	1.02
	6.5–8.5 months	1.78	(0.93–3.42)			1.64	(0.85–3.16)			2.16	(1.23–3.80)			2.02	(1.14–3.56)		

*Abbreviations* MRI = magnetic resonance imaging; OR = odds ratio; CI = confidence interval; VIF = variance inflation factor; GTV = gross tumor volume

### Dose for GTV, LF, and ARE

GTV (cc) was a risk factor for LF (Table 2). GTV (cc) exhibited no correlation between GTV D80 greater or smaller than 42 Gy (*τ* = 0.21) and D98 greater or smaller than 39 Gy (*τ* = 0.22). For GTV > 1 cc, 87 brain metastases (69.6%) were GTV D80 > 42 Gy and 90 brain metastases (72.0%) were GTV D98 > 39 Gy.

**Fig. 2:**
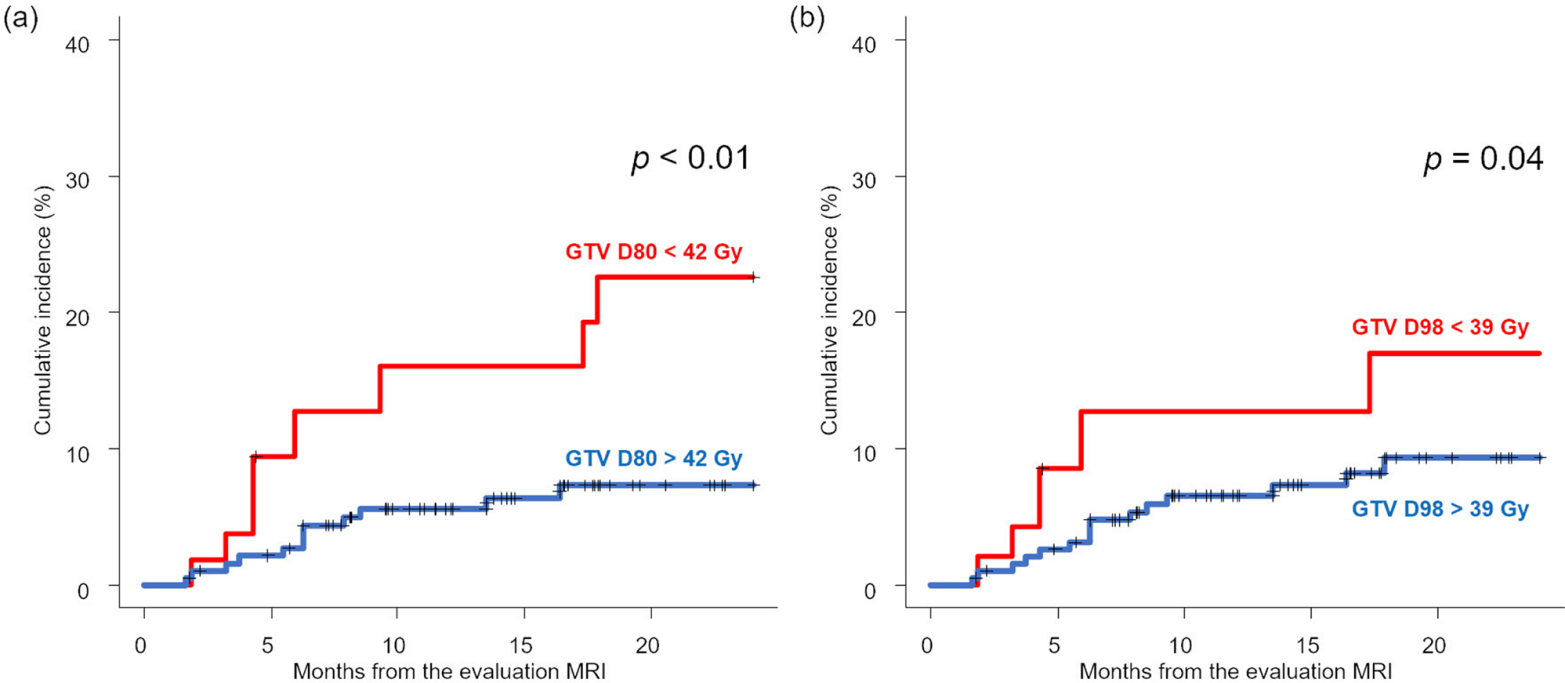
Local failure rate comparisons based on GTV dose in five fractions. (**a**) Under vs. over GTV D80 42 Gy; (**b**) D98 39 Gy. GTV = gross tumor volume

D98 > 39 Gy was not a prognostic factor for LF in multivariate analysis (Table 2); however, D80 > 42 Gy was a significant prognostic factor for LF (HR, 0.37; *p* = 0.01; Table 2). The LF at 0.5 and 1.5 years from the MRI evaluation (approximately 1 and 2 years from fSRS) was 2.70 and 7.31%, respectively, for D80 > 42 Gy (Fig. 2).

The ARE at 1 and 2 years from fSRS was 6.01% and 13.14%, respectively. D80 > 42 Gy and D98 > 39 Gy were not a prognostic factor for ARE (Supplementary Tables 4 and Supplementary Figure).

## Discussion

To our knowledge, this is the first study to report that GTV D80 > 42 Gy in five fractions is a significant prognostic factor for both tumor volume reduction and LF in Linac fSRS for brain metastases > 0.3 cc. Prescribed dose has been established as a predictive factor for LF [5, 24]. A higher prescribed dose is associated with an improved local control trend [4, 5, 24]. Moreover, a prescribed dose of > 30 Gy in five fractions was a predictive factor for LF [3, 11, 14]. However, different isodose and PTV margins result in different doses for the GTV even with the same prescribed dose [4]. Few studies have reported the GTV dose and LF [9]. This is the first study to discuss GTV dose and LF treated by fSRS in five fractions. Further studies incorporating GTV dose and LF are needed.

As previously recommended by the International Commission on Radiation Units and Measurements (ICRU), the dose in the PTV should be confined within 95–107% of the prescribed dose [25]; therefore, 80–90% isodose was used for patients undergoing Linac fSRS [3, 12, 24]. In this study, achieving a GTV D80 > 42 Gy when prescribed 35 Gy (the median dose) with 80% isodose was almost impossible, and an inhomogeneous distribution was needed. Inhomogeneous distribution has been reported to predict good local control in gamma knife SRS [26, 27] and may lead to better tumor volume reduction and LF.

Some problems are associated with LF in fSRS for brain metastases. First, many fSRS characteristics have underpowered significance because many patients die from extracranial disease. Second, tumor recurrence and brain necrosis are often difficult to diagnose. In cases with volume progression after fSRS, viable tumor tissue and brain necrotic change are often mixed on pathological evaluation [11, 28]. Misdiagnoses of tumor recurrence and brain necrosis, evaluated only using imaging, are unavoidable. In this study, O65R and O90R 6 months after fSRS were prognostic factors for LF. This was consistent with the results of our previous study [19]. O65R in brain metastases corresponds to a partial response according to the RANO-BM guidelines [15]. The median volume reduction rates were 44.2% at 3 months and 69.6% at 6 months after SRS and fSRS [17]. A partial response at 6 months after fSRS may be the first step toward long-term local control.

In this study, GTV D80 and GTV D98 were more predictive of volume reduction than GTV D2. Increasing the GTV D80 dose predicted good local control. Lucia et al. reported that an inhomogeneous dose distribution was a significant prognostic factor for local control compared to a homogeneous distribution with the same PTV dose; however, D2 was not a significant prognostic factor for local control [29]. These results may seem contradictory; however, GTV D80 and GTV D98 might be higher in inhomogeneous dose distributions, leading to good local control. Inhomogeneous dose distribution should be used to increase mainly GTV D80 and GTV D98.

GTV D80 was the most predictive for volume reduction in this study and our previous one [19]. The HyTEC group recommends that the maximum (D2) and peripheral (D98) doses for GTV should be included when reporting outcomes [4]. This study indicates that reporting only GTV D98 may be insufficient. A more inhomogeneous dose distribution can now be easily obtained with the advancement in Linac fSRS, including non-coplanar VMAT [30].

Our study has some limitations. First, it was retrospective. Second, the median tumor volume was 1.1 cc. However, we excluded brain metastases < 0.3 cc because volumetric measurements are unsuitable for very small brain metastases. The optimal GTV dose for very large brain metastases should be evaluated in future studies. Third, the effects of systemic therapy were not analyzed because this study included a variety of primary cancers. When reporting the same number of fractions in fSRS for brain metastases, the number of analyzed brain metastases is often small. The strength of our study is that this is one of the largest studies of its kind, with 241 brain metastases treated with five fractions and a relatively long follow-up period.

## Conclusions

GTV D80 was the most predictive factor for volume reduction after fSRS. GTV D80 > 42 Gy in five fractions is required for good volume reduction and local control. The goal of planning an inhomogeneous dose distribution for fSRS in brain metastases is to increase the GTV D80 and GTV D98. An inhomogeneous dose distribution can now be easily obtained with the advancement of Linac fSRS, including non-coplanar VMAT. Further studies on inhomogeneous dose distributions are required.

### Electronic supplementary material

Below is the link to the electronic supplementary material.

Supplementary Material 1

Supplementary Material 2

Supplementary Material 3

Supplementary Material 4

Supplementary Material 5

## Data Availability

The data that support the findings of this study are available upon request from the corresponding author. The data are not publicly available because of privacy or ethical restrictions.

## Abbreviations

AICc: Akaike information criterion
ARE: Adverse Radiation Effect
EVRs: Evidence ratios
fSRS: Fractionated stereotactic radiosurgery
GTV: Gross tumor volume
HR: Hazard ratios
LF: Local failure
MRI: Magnetic resonance imaging
OR: Odds ratios
PTV: Planning target volume
VIF: Variance inflation factor
