# Supplementary material for: Association of increasing gross tumor volume dose with tumor volume reduction and local control in fractionated stereotactic radiosurgery for unresected brain metastases
Source: Radiat Oncol. 2024 Jul 27;19:95. doi: 10.1186/s13014-024-02487-6 (PMC11282845; doi:10.1186/s13014-024-02487-6)
Supplement: Supplementary file 1 — Supplementary Material 1 [file 13014_2024_2487_MOESM1_ESM.docx]

| **Supplementary Table 1. Univariate analysis results for over 65% and 90% volume reduction at the MRI evaluation** | | | | | | | |
| --- | --- | --- | --- | --- | --- | --- | --- |
|  |  | Over 65% volume reduction | | | Over 90% volume reduction | | |
|  |  | OR | (95% CI) | *p*-value | OR | (95% CI) | *p*-value |
| Age (years) | 22–65 | 1 |  | 0.11 | 1 |  | 0.48 |
|  | >65 | 0.62 | (0.35–1.11) |  | 0.83 | (0.50–1.39) |  |
| PS | 0–1 | 1 |  | 0.14 | 1 |  | 0.14 |
|  | 2–3 | 0.65 | (0.36–1.16) |  | 0.68 | (0.41–1.13) |  |
| Primary cancer | Lung and Breast | 1 |  | 0.46 | 1 |  | 0.17 |
|  | Others | 1.30 | (0.65–2.62) |  | 0.66 | (0.36–1.19) |  |
| Each GTV (cc) | 0.3–1 | 1 |  | 0.26 | 1 |  | 0.22 |
|  | >1 | 0.71 | (0.40–1.28) |  | 0.73 | (0.44–1.21) |  |
| Time for the evaluation MRI (months) | 5–6.5 | 1 |  | 0.16 | 1 |  | 0.02 |
|  | 6.5–8.5 | 1.56 | (0.84–2.89) |  | 1.87 | (1.10–3.17) |  |
| GTV dose | D98 | 1.08 | (1.02–1.14) | < 0.01 | 1.08 | (1.03–1.14) | < 0.01 |
|  | D80 | 1.07 | (1.02–1.13) | < 0.01 | 1.07 | (1.02–1.12) | < 0.01 |
|  | D60 | 1.05 | (1.01–1.10) | 0.01 | 1.05 | (1.01–1.09) | 0.01 |
|  | D40 | 1.04 | (1.01–1.08) | 0.02 | 1.04 | (1.01–1.07) | 0.02 |
|  | D20 | 1.04 | (1.01–1.07) | 0.02 | 1.03 | (1.00–1.06) | 0.03 |
|  | D2 | 1.03 | (1.00–1.06) | 0.03 | 1.03 | (1.00–1.05) | 0.04 |
| GTV dose | D80 < 42 Gy | 1 |  |  | 1 |  |  |
|  | D80 > 42 Gy | 2.93 | (1.53–5.60) | < 0.01 | 3.68 | (1.87–7.23) | < 0.01 |
| GTV dose | D98 < 39 Gy | 1 |  |  | 1 |  |  |
|  | D98 > 39 Gy | 3.81 | (1.95–7.45) | < 0.01 | 4.95 | (2.33–10.52) | < 0.01 |

Abbreviations: MRI = Magnetic resonance imaging; OR = odds ratio; CI = confidence interval; PS = performance status; GTV = gross tumor volume; For Gy variables OR, increase per 1
