# Supplementary material for: Association of increasing gross tumor volume dose with tumor volume reduction and local control in fractionated stereotactic radiosurgery for unresected brain metastases
Source: Radiat Oncol. 2024 Jul 27;19:95. doi: 10.1186/s13014-024-02487-6 (PMC11282845; doi:10.1186/s13014-024-02487-6)
Supplement: Supplementary file 2 — Supplementary Material 2 [file 13014_2024_2487_MOESM2_ESM.docx]

| **Supplementary Table 2. AICc and EVR value for over 65% and 90% volume reduction at MRI evaluation** | | | | | | |
| --- | --- | --- | --- | --- | --- | --- |
|  |  |  | Over 65% volume reduction | | Over 90% volume reduction | |
| Model variables | |  | AICc | EVR | AICc | EVR |
| Base = Null |  |  |  |  |  |  |
|  | Base + GTV dose | D98 | 270.013 | 16.004 | 327.110 | 267.032 |
|  |  | D80 | 268.790 | 8.683 | 326.936 | 244.782 |
|  |  | D60 | 270.457 | 19.983 | 329.276 | 788.686 |
|  |  | D40 | 271.922 | 41.570 | 330.401 | 1384.187 |
|  |  | D20 | 270.938 | 25.416 | 330.963 | 1833.291 |
|  |  | D2 | 272.063 | 44.606 | 331.787 | 2767.966 |
| Base = Age (22–65, >65) |  |  |  |  |  |  |
|  | Base |  | 274.217 | 130.957 | 335.611 | 18729.745 |
|  | Base + GTV dose | D98 | 266.586 | 2.886 | 327.183 | 277.053 |
|  |  | D80 | 265.740 | **1.890** | 327.333 | 298.631 |
|  |  | D60 | 268.108 | 6.176 | 330.013 | 1140.486 |
|  |  | D40 | 268.854 | 8.968 | 331.301 | 2171.580 |
|  |  | D20 | 269.216 | 10.748 | 332.038 | 3139.159 |
|  |  | D2 | 270.640 | 21.905 | 332.970 | 5002.585 |
| Base = PS (0–1, 2–3) |  |  |  |  |  |  |
|  | Base |  | 274.692 | 166.063 | 333.910 | 8001.371 |
|  | Base + GTV dose | D98 | 271.359 | 31.381 | 328.490 | 532.568 |
|  |  | D80 | 270.211 | 17.676 | 328.294 | 482.852 |
|  |  | D60 | 271.565 | 34.786 | 330.306 | 1320.427 |
|  |  | D40 | 271.814 | 39.398 | 331.186 | 2050.236 |
|  |  | D20 | 271.655 | 36.387 | 331.566 | 2479.247 |
|  |  | D2 | 272.527 | 56.273 | 332.187 | 3381.958 |
| Base = Primary cancer (Lung and Breast, others) | |  |  |  |  |  |
|  | Base |  | 276.270 | 42.092 | 334.186 | 9185.378 |
|  | Base + GTV dose | D98 | 272.045 | 44.221 | 323.699 | 48.531 |
|  |  | D80 | 270.822 | 23.992 | 323.829 | 51.791 |
|  |  | D60 | 272.459 | 54.392 | 326.975 | 249.687 |
|  |  | D40 | 272.885 | 67.303 | 328.608 | 564.935 |
|  |  | D20 | 272.850 | 66.136 | 329.572 | 914.804 |
|  |  | D2 | 273.850 | 109.039 | 330.979 | 1848.648 |
| Base = Volume (0.3–1 cc, >1 cc) |  |  |  |  |  |  |
|  | Base |  | 275.551 | 255.154 | 334.602 | 11309.159 |
|  | Base + GTV dose | D98 | 271.597 | 35.347 | 328.610 | 565.500 |
|  |  | D80 | 270.140 | 17.060 | 328.043 | 425.902 |
|  |  | D60 | 271.563 | 34.751 | 330.053 | 1163.526 |
|  |  | D40 | 271.932 | 41.792 | 331.092 | 1956.104 |
|  |  | D20 | 272.020 | 43.672 | 331.742 | 2707.308 |
|  |  | D2 | 273.225 | 79.775 | 332.688 | 4344.692 |
| Base = Time for MRI evaluation (5–6.5 months, 6.5–8.5 months) | |  |  |  |  |  |
|  | Base |  | 274.794 | 174.752 | 330.626 | 1549.005 |
|  | Base + GTV dose | D98 | 270.277 | 18.269 | 323.567 | 45.432 |
|  |  | D80 | 268.611 | 7.942 | 322.553 | 27.364 |
|  |  | D60 | 269.946 | 15.482 | 324.602 | 76.227 |
|  |  | D40 | 270.211 | 17.676 | 325.613 | 126.369 |
|  |  | D20 | 269.984 | 15.779 | 326.008 | 153.963 |
|  |  | D2 | 271.094 | 27.487 | 326.884 | 238.581 |
| Base = Age + PS | |  |  |  |  |  |
|  | Base |  | 274.206 | 130.283 | 335.495 | 17680.367 |
|  | Base + GTV dose | D98 | 268.154 | 6.319 | 328.753 | 607.367 |
|  |  | D80 | 267.295 | 4.113 | 328.842 | 635.005 |
|  |  | D60 | 269.306 | 11.242 | 331.179 | 2042.915 |
|  |  | D40 | 269.806 | 14.435 | 332.209 | 3419.101 |
|  |  | D20 | 269.971 | 15.676 | 332.745 | 4469.952 |
|  |  | D2 | 271.163 | 28.450 | 333.465 | 6406.913 |
| Base = Age + Primary cancer | |  |  |  |  |  |
|  | Base |  | 275.892 | 302.690 | 335.490 | 17636.221 |
|  | Base + GTV dose | D98 | 268.518 | 7.581 | 322.257 | 23.597 |
|  |  | D80 | 267.701 | 5.039 | 323.008 | 34.351 |
|  |  | D60 | 270.148 | 17.127 | 326.822 | 231.281 |
|  |  | D40 | 270.905 | 25.006 | 328.793 | 619.636 |
|  |  | D20 | 271.247 | 29.670 | 330.129 | 1208.497 |
|  |  | D2 | 272.594 | 58.185 | 331.745 | 2711.163 |
| Base = Age + Volume | |  |  |  |  |  |
|  | Base |  | 274.978 | 191.658 | 336.148 | 24506.902 |
|  | Base + GTV dose | D98 | 268.327 | 6.890 | 328.786 | 617.471 |
|  |  | D80 | 267.189 | 3.901 | 328.505 | 536.536 |
|  |  | D60 | 269.274 | 11.063 | 330.835 | 1720.092 |
|  |  | D40 | 269.921 | 15.289 | 332.031 | 3127.949 |
|  |  | D20 | 270.357 | 19.013 | 332.858 | 4729.775 |
|  |  | D2 | 271.874 | 40.595 | 333.917 | 8031.551 |
| Base = Age + Time for MRI evaluation | |  |  |  |  |  |
|  | Base |  | 273.821 | 107.470 | 331.874 | 2892.019 |
|  | Base + GTV dose | D98 | 266.031 | **2.186** | 323.111 | 36.166 |
|  |  | D80 | 264.467 | **1.000** | 322.354 | 24.770 |
|  |  | D60 | 266.487 | 2.746 | 324.747 | 81.952 |
|  |  | D40 | 267.014 | 3.574 | 325.938 | 148.656 |
|  |  | D20 | 267.164 | 3.852 | 326.549 | 201.771 |
|  |  | D2 | 268.750 | 8.513 | 327.578 | 337.523 |
| Base = PS + Primary cancer | |  |  |  |  |  |
|  | Base |  | 275.835 | 294.185 | 334.456 | 10516.598 |
|  | Base + GTV dose | D98 | 273.365 | 85.553 | 325.635 | 127.757 |
|  |  | D80 | 272.222 | 48.310 | 325.724 | 133.571 |
|  |  | D60 | 273.479 | 90.571 | 328.602 | 563.199 |
|  |  | D40 | 273.651 | 98.705 | 329.988 | 1126.232 |
|  |  | D20 | 273.405 | 87.281 | 330.770 | 1665.087 |
|  |  | D2 | 274.087 | 122.748 | 331.894 | 2920.859 |
| Base = PS + Volume | |  |  |  |  |  |
|  | Base |  | 275.557 | 256.008 | 334.567 | 11116.770 |
|  | Base + GTV dose | D98 | 272.945 | 69.348 | 330.004 | 1135.278 |
|  |  | D80 | 271.595 | 35.309 | 329.442 | 857.167 |
|  |  | D60 | 272.739 | 62.560 | 331.164 | 2027.650 |
|  |  | D40 | 272.917 | 68.384 | 331.977 | 3044.625 |
|  |  | D20 | 272.833 | 65.571 | 332.445 | 3847.323 |
|  |  | D2 | 273.785 | 105.545 | 333.186 | 5572.689 |
| Base = PS + Time for MRI evaluation | |  |  |  |  |  |
|  | Base |  | 274.399 | 143.482 | 329.961 | 1111.216 |
|  | Base + GTV dose | D98 | 271.485 | 33.419 | 324.621 | 76.948 |
|  |  | D80 | 269.929 | 15.350 | 323.614 | 46.508 |
|  |  | D60 | 270.940 | 25.448 | 325.321 | 109.195 |
|  |  | D40 | 270.978 | 25.936 | 326.061 | 158.085 |
|  |  | D20 | 270.554 | 20.981 | 326.250 | 173.753 |
|  |  | D2 | 271.469 | 33.153 | 326.887 | 238.921 |
| Base = Primary cancer + Volume | |  |  |  |  |  |
|  | Base |  | 276.852 | 489.170 | 334.969 | 13591.639 |
|  | Base + GTV dose | D98 | 273.633 | 97.820 | 325.576 | 124.044 |
|  |  | D80 | 272.177 | 47.235 | 325.361 | 111.401 |
|  |  | D60 | 273.541 | 93.423 | 328.165 | 452.656 |
|  |  | D40 | 273.856 | 109.359 | 329.685 | 967.904 |
|  |  | D20 | 273.877 | 110.513 | 330.741 | 1641.118 |
|  |  | D2 | 274.933 | 187.379 | 332.201 | 3405.452 |
| Base = Primary cancer + Time for MRI evaluation | |  |  |  |  |  |
|  | Base |  | 276.424 | 394.931 | 330.026 | 1147.924 |
|  | Base + GTV dose | D98 | 272.319 | 50.710 | 318.903 | 4.411 |
|  |  | D80 | 270.640 | 21.903 | 317.962 | 2.756 |
|  |  | D60 | 271.996 | 43.148 | 320.837 | 11.601 |
|  |  | D40 | 272.259 | 49.212 | 322.413 | 25.512 |
|  |  | D20 | 272.017 | 43.603 | 323.300 | 39.751 |
|  |  | D2 | 273.061 | 73.489 | 324.899 | 88.423 |
| Base = Volume + Time for MRI evaluation | |  |  |  |  |  |
|  | Base |  | 275.721 | 277.886 | 331.463 | 2354.798 |
|  | Base + GTV dose | D98 | 271.956 | 42.293 | 325.264 | 106.127 |
|  |  | D80 | 270.106 | 16.771 | 323.956 | 55.182 |
|  |  | D60 | 271.242 | 29.596 | 325.728 | 133.838 |
|  |  | D40 | 271.432 | 32.545 | 326.674 | 214.784 |
|  |  | D20 | 271.283 | 30.209 | 327.166 | 274.687 |
|  |  | D2 | 272.472 | 54.742 | 328.161 | 451.752 |
| Base = Age + PS + Primary cancer | |  |  |  |  |  |
|  | Base |  | 275.633 | 265.903 | 335.859 | 21207.982 |
|  | Base + GTV dose | D98 | 270.180 | 17.405 | 324.314 | 66.006 |
|  |  | D80 | 269.338 | 11.424 | 325.025 | 94.184 |
|  |  | D60 | 271.368 | 31.524 | 328.605 | 564.108 |
|  |  | D40 | 271.832 | 39.755 | 330.330 | 1336.415 |
|  |  | D20 | 271.928 | 41.710 | 331.435 | 2322.143 |
|  |  | D2 | 272.967 | 70.123 | 332.791 | 4574.468 |
| Base = Age + PS + Volume | |  |  |  |  |  |
|  | Base |  | 275.154 | 209.272 | 336.178 | 24875.357 |
|  | Base + GTV dose | D98 | 269.923 | 15.306 | 330.367 | 1361.369 |
|  |  | D80 | 268.805 | 8.752 | 330.051 | 1162.404 |
|  |  | D60 | 270.581 | 21.269 | 332.076 | 3199.487 |
|  |  | D40 | 271.009 | 26.344 | 333.034 | 5165.441 |
|  |  | D20 | 271.251 | 29.733 | 333.660 | 7063.852 |
|  |  | D2 | 272.533 | 56.444 | 334.504 | 10772.434 |
| Base = Age + PS + Time for MRI evaluation | |  |  |  |  |  |
|  | Base |  | 267.470 | 4.488 | 331.286 | 2155.187 |
|  | Base + GTV dose | D98 | 267.549 | 4.670 | 324.436 | 70.158 |
|  |  | D80 | 265.997 | **2.150** | 323.635 | 47.005 |
|  |  | D60 | 267.644 | 4.898 | 325.662 | 129.508 |
|  |  | D40 | 267.901 | 5.569 | 326.564 | 203.313 |
|  |  | D20 | 267.816 | 5.337 | 326.941 | 245.487 |
|  |  | D2 | 269.114 | 10.214 | 327.711 | 360.772 |
| Base = Age + Primary cancer + Volume | |  |  |  |  |  |
|  | Base |  | 276.516 | 413.490 | 336.317 | 26665.688 |
|  | Base + GTV dose | D98 | 270.328 | 18.742 | 324.243 | 63.704 |
|  |  | D80 | 269.217 | 10.754 | 324.644 | 77.847 |
|  |  | D60 | 271.342 | 31.117 | 328.095 | 437.137 |
|  |  | D40 | 271.973 | 42.659 | 329.941 | 1100.198 |
|  |  | D20 | 272.363 | 51.844 | 331.334 | 2207.787 |
|  |  | D2 | 273.770 | 104.768 | 333.032 | 5160.278 |
| Base = Age + Primary cancer + Time for MRI evaluation | |  |  |  |  |  |
|  | Base |  | 275.629 | 265.372 | 330.919 | 1793.874 |
|  | Base + GTV dose | D98 | 267.853 | 5.437 | 316.364 | **1.240** |
|  |  | D80 | 266.268 | **2.461** | 315.935 | **1.000** |
|  |  | D60 | 268.441 | 7.295 | 319.542 | 6.072 |
|  |  | D40 | 269.033 | 9.808 | 321.538 | 16.473 |
|  |  | D20 | 269.222 | 10.781 | 322.896 | 32.484 |
|  |  | D2 | 270.809 | 23.837 | 324.861 | 86.769 |
| Base = Age + Volume + Time for MRI evaluation | |  |  |  |  |  |
|  | Base |  | 274.769 | 172.627 | 332.749 | 4478.901 |
|  | Base + GTV dose | D98 | 267.856 | 5.445 | 324.905 | 88.699 |
|  |  | D80 | 266.057 | **2.215** | 323.831 | 51.844 |
|  |  | D60 | 267.843 | 5.410 | 325.934 | 148.375 |
|  |  | D40 | 268.288 | 6.758 | 327.055 | 259.887 |
|  |  | D20 | 268.519 | 7.586 | 327.761 | 369.905 |
|  |  | D2 | 270.198 | 17.562 | 328.910 | 657.041 |
| Base = PS + Primary cancer + Volume | |  |  |  |  |  |
|  | Base |  | 276.557 | 422.054 | 335.339 | 16352.448 |
|  | Base + GTV dose | D98 | 274.919 | 186.093 | 327.519 | 327.748 |
|  |  | D80 | 273.581 | 95.321 | 327.272 | 289.671 |
|  |  | D60 | 274.602 | 158.816 | 329.828 | 1039.761 |
|  |  | D40 | 274.691 | 166.043 | 331.118 | 1981.770 |
|  |  | D20 | 274.509 | 151.600 | 331.964 | 3025.240 |
|  |  | D2 | 275.254 | 220.026 | 333.171 | 5531.674 |
| Base = PS + Primary cancer + Time for MRI evaluation | |  |  |  |  |  |
|  | Base |  | 275.853 | 296.822 | 329.929 | 1093.494 |
|  | Base + GTV dose | D98 | 273.548 | 93.761 | 320.720 | 10.944 |
|  |  | D80 | 271.997 | 43.174 | 319.747 | 6.728 |
|  |  | D60 | 272.987 | 70.827 | 322.322 | 24.380 |
|  |  | D40 | 272.997 | 71.182 | 323.621 | 46.677 |
|  |  | D20 | 272.537 | 56.557 | 324.268 | 64.505 |
|  |  | D2 | 273.236 | 80.218 | 325.572 | 123.810 |
| Base = Primary cancer + Volume + Time for MRI evaluation | |  |  |  |  |  |
|  | Base |  | 277.227 | 590.006 | 331.165 | 2028.664 |
|  | Base + GTV dose | D98 | 274.024 | 118.955 | 320.923 | 12.113 |
|  |  | D80 | 272.171 | 47.099 | 319.778 | 6.833 |
|  |  | D60 | 273.305 | 83.034 | 322.396 | 25.299 |
|  |  | D40 | 273.477 | 90.491 | 323.887 | 53.316 |
|  |  | D20 | 273.300 | 82.826 | 324.837 | 85.734 |
|  |  | D2 | 274.397 | 143.344 | 326.504 | 197.304 |
| Base = Age + PS + Primary cancer + Volume | |  |  |  |  |  |
|  | Base |  | 276.440 | 398.117 | 336.780 | 33615.608 |
|  | Base + GTV dose | D98 | 271.995 | 43.127 | 326.315 | 179.497 |
|  |  | D80 | 270.886 | 24.771 | 326.678 | 215.220 |
|  |  | D60 | 272.640 | 59.541 | 329.908 | 1082.101 |
|  |  | D40 | 273.008 | 71.569 | 331.524 | 2427.603 |
|  |  | D20 | 273.159 | 77.181 | 332.689 | 4346.645 |
|  |  | D2 | 274.261 | 133.909 | 334.127 | 8920.967 |
| Base = Age + PS + Primary cancer + Time for MRI evaluation | | | |  |  |  |
|  | Base |  | 275.240 | 218.491 | 330.932 | 1805.776 |
|  | Base + GTV dose | D98 | 269.496 | 12.362 | 318.403 | 3.435 |
|  |  | D80 | 267.925 | 5.636 | 317.921 | 2.700 |
|  |  | D60 | 269.691 | 13.628 | 321.257 | 14.313 |
|  |  | D40 | 269.981 | 15.755 | 322.971 | 33.722 |
|  |  | D20 | 269.899 | 15.122 | 324.059 | 58.100 |
|  |  | D2 | 271.137 | 28.083 | 325.712 | 132.775 |
| Base = PS + Primary cancer + Volume + Time for MRI evaluation | | | |  |  |  |
|  | Base |  | 276.786 | 473.308 | 331.165 | 2028.893 |
|  | Base + GTV dose | D98 | 275.224 | 216.732 | 322.752 | 30.225 |
|  |  | D80 | 273.530 | 92.913 | 321.581 | 16.830 |
|  |  | D60 | 274.330 | 138.609 | 323.913 | 54.009 |
|  |  | D40 | 274.275 | 134.850 | 325.144 | 99.949 |
|  |  | D20 | 273.887 | 111.070 | 325.859 | 142.902 |
|  |  | D2 | 274.646 | 162.335 | 327.233 | 284.053 |
| Base = Age + PS, Primary cancer, Volume, Time for MRI evaluation | | | |  |  |  |
|  | Base |  | 276.255 | 362.912 | 332.224 | 3444.932 |
|  | Base + GTV dose | D98 | 271.419 | 32.330 | 320.503 | 9.816 |
|  |  | D80 | 269.652 | 13.363 | 319.860 | 7.117 |
|  |  | D60 | 271.194 | 28.890 | 322.951 | 33.381 |
|  |  | D40 | 271.407 | 32.136 | 324.587 | 75.640 |
|  |  | D20 | 271.387 | 31.817 | 325.730 | 133.953 |
|  |  | D2 | 272.682 | 60.794 | 327.446 | 315.919 |

Abbreviations: AICc = Akaike Information Criterion; EVR = Evidence ratios; MRI= Magnetic resonance imaging; GTV = gross tumor volume; PS = performance status
