# Supplementary material for: Association of increasing gross tumor volume dose with tumor volume reduction and local control in fractionated stereotactic radiosurgery for unresected brain metastases
Source: Radiat Oncol. 2024 Jul 27;19:95. doi: 10.1186/s13014-024-02487-6 (PMC11282845; doi:10.1186/s13014-024-02487-6)
Supplement: Supplementary file 3 — Supplementary Material 3 [file 13014_2024_2487_MOESM3_ESM.docx]

| **Supplementary Table 3. Multivariate analysis results for over 65% and 90% volume reduction at MRI evaluation** | | | | | | | | | | | | | | | | | |
| --- | --- | --- | --- | --- | --- | --- | --- | --- | --- | --- | --- | --- | --- | --- | --- | --- | --- |
|  |  | Over 65% volume reduction | | |  | Over 65% volume reduction | | |  | Over 90% volume reduction | | |  | Over 90% volume reduction | | |  |
|  |  | OR | (95% CI) | *p*-value | VIF | OR | (95% CI) | *p*-value | VIF | OR | (95% CI) | *p*-value | VIF | OR | (95% CI) | *p*-value | VIF |
| GTV dose | D80 | 1.09 | (1.04–1.15) | <0.01 | 1.04 |  |  |  |  | 1.11 | (1.05–1.16) | <0.01 | 1.07 |  |  |  |  |
|  | D98 |  |  |  |  | 1.10 | (1.03–1.16) | <0.01 | 1.05 |  |  |  |  | 1.12 |  | <0.01 | 1.16 |
| Age (years) | 22–65 | 1 |  | 0.01 | 1.04 | 1 |  | 0.01 | 1.06 | 1 |  | 0.05 | 1.11 | 1 |  | 0.03 | 1.09 |
|  | >65 | 0.45 | (0.24–0.85) |  |  | 0.45 | (0.24–0.85) |  |  | 0.56 | (0.32–0.99) |  |  | 0.54 | (0.31–0.96) |  |  |
| Primary cancer | Lung and Breast |  |  |  |  |  |  |  |  | 1 |  | <0.01 | 1.02 | 1 |  | <0.01 | 1.13 |
|  | Others |  |  |  |  |  |  |  |  | 0.38 | (0.19–0.74) |  |  | 0.37 | (0.19–0.72) |  |  |
| Time for MRI evaluation | 5–6.5 months | 1 |  | 0.08 | 1.01 | 1 |  | 0.11 | 1.01 | 1 |  | <0.01 | 1.13 | 1 |  | <0.01 | 1.02 |
|  | 6.5–8.5 months | 1.80 | (0.94–3.44) |  |  | 1.68 | (0.89–3.19) |  |  | 2.36 | (1.34–4.16) |  |  | 2.22 | (1.26–3.88) |  |  |

Abbreviations: MRI= Magnetic resonance imaging; OR = odds ratio; CI = confidence interval; VIF = variance inflation factor; GTV = gross tumor volume
