# Supplementary material for: Association of increasing gross tumor volume dose with tumor volume reduction and local control in fractionated stereotactic radiosurgery for unresected brain metastases
Source: Radiat Oncol. 2024 Jul 27;19:95. doi: 10.1186/s13014-024-02487-6 (PMC11282845; doi:10.1186/s13014-024-02487-6)
Supplement: Supplementary file 4 — Supplementary Material 4 [file 13014_2024_2487_MOESM4_ESM.docx]

| **Supplementary Table 4. Results of the univariable and multivariate analyses of adverse radiation effect** | | | | | | | | | | | | | | |
| --- | --- | --- | --- | --- | --- | --- | --- | --- | --- | --- | --- | --- | --- | --- |
|  |  | Univariate | | | Multivariate | | | Multivariate | | | Multivariate | | |  |
|  |  | HR | (95% CI) | *p*-value | HR | (95% CI) | *p*-value | HR | (95% CI) | *p*-value | HR | (95% CI) | *p*-value |  |
| each GTV (cc) | 0.3–1 | 1 |  | <0.01 | 1 |  | <0.01 | 1 |  | <0.01 | 1 |  | <0.01 |  |
|  | >1 | 4.57 | (1.85–11.27) |  | 4.85 | (1.99–11.79) |  | 4.60 | (1.83–11.52) |  | 4.39 | (1.75–11.05) |  |  |
| Age (years) | 22–65 | 1 |  | 0.84 |  |  |  |  |  |  |  |  |  |  |
|  | >65 | 0.93 | (0.47–1.86) |  |  |  |  |  |  |  |  |  |  |  |
| PS | 0–1 | 1 |  | <0.01 | 1 |  | <0.01 |  |  |  |  |  |  |  |
|  | 2–3 | 0.24 | (0.10–0.64) |  | 0.23 | (0.09–0.59) |  |  |  |  |  |  |  |  |
| Primary cancer | Lung and breast | 1 |  | 0.81 |  |  |  |  |  |  |  |  |  |  |
|  | Others | 0.90 | (0.39–2.10) |  |  |  |  |  |  |  |  |  |  |  |
| GTV dose | D80 < 42 Gy | 1 |  | 0.44 |  |  |  | 1 |  | 0.92 |  |  |  |  |
|  | D80 > 42 Gy | 0.74 | (0.34–1.59) |  |  |  |  | 1.04 | (0.48–2.27) |  |  |  |  |  |
| GTV dose | D98 < 39 Gy | 1 |  | 0.15 |  |  |  |  |  |  | 1 |  | 0.62 |  |
|  | D98 > 39 Gy | 0.56 | (0.26–1.22) |  |  |  |  |  |  |  | 0.82 | (0.37–1.83) |  |  |

Abbreviations: HR = hazard ratio; CI = confidence interval; GTV = gross tumor volume; PS = performance status
