# Supplementary material for: Association of increasing gross tumor volume dose with tumor volume reduction and local control in fractionated stereotactic radiosurgery for unresected brain metastases
Source: Radiat Oncol. 2024 Jul 27;19:95. doi: 10.1186/s13014-024-02487-6 (PMC11282845; doi:10.1186/s13014-024-02487-6)

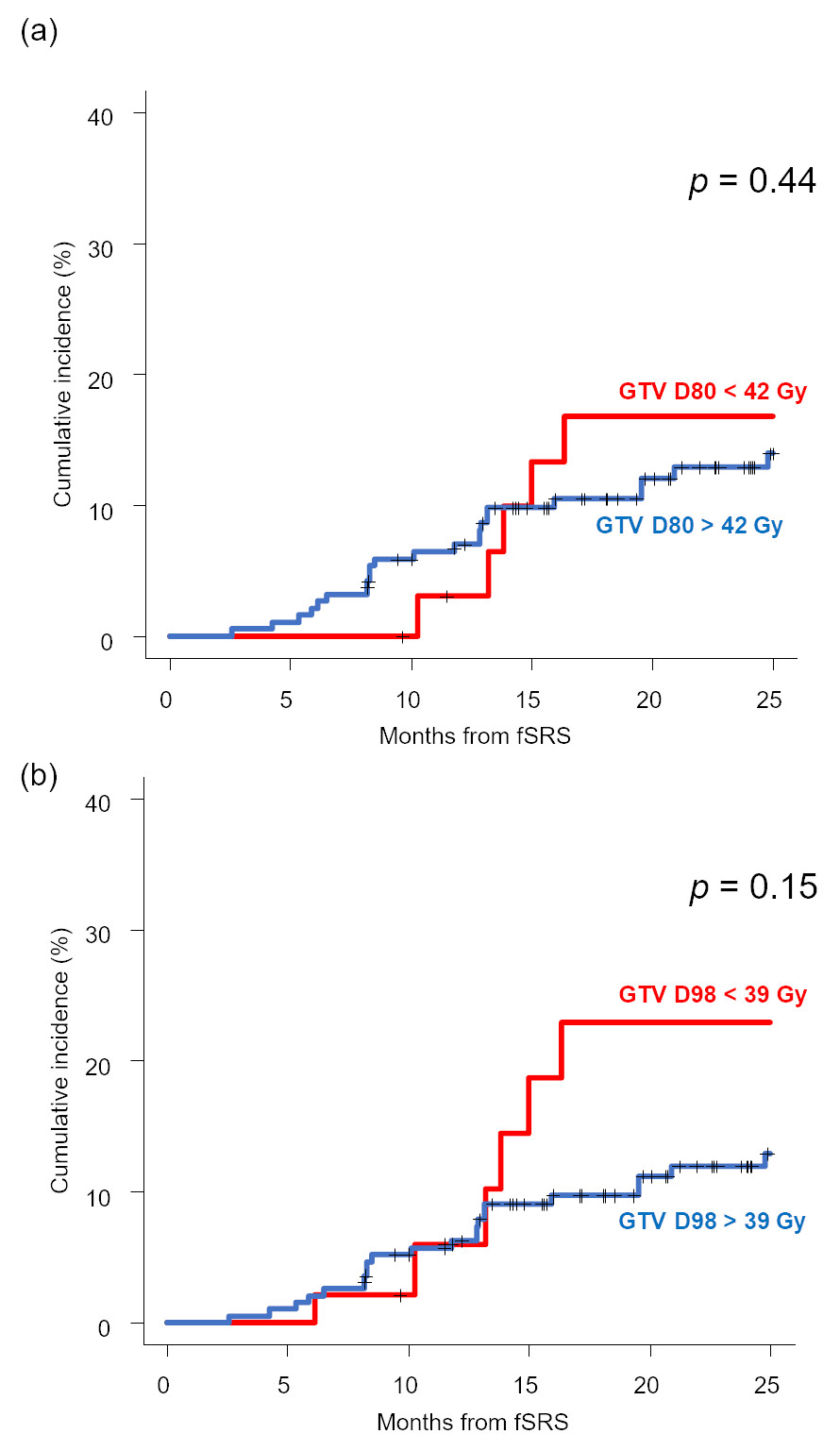
Supplementary Figure:

Adverse Radiation Effect rate comparisons based on GTV dose in five fractions

(a) Under vs. over GTV D80 42 Gy; (b) D98 39 Gy.

GTV = gross tumor volume


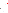

Supplement: Supplementary file 5 — Supplementary Material 5 [file 13014_2024_2487_MOESM5_ESM.docx]
